# Differential regulation of sleep by blue, green, and red light in *Drosophila melanogaster*

**DOI:** 10.3389/fnbeh.2024.1476501

**Published:** 2024-10-30

**Authors:** Samuel M. Bond, Aaliyah J. Peralta, Dilhan Sirtalan, Dominic A. Skeele, Haoyang Huang, Debra R. Possidente, Christopher G. Vecsey

**Affiliations:** ^1^Neuroscience Program, Skidmore College, Saratoga Springs, NY, United States; ^2^Department of Neurobiology, UMass Chan Medical School, Worcester, MA, United States

**Keywords:** sleep, *Drosophila melanogaster*, light color, red, blue, green

## Abstract

**Introduction:**

Exposure to blue-enriched light from electronic devices is an emergent disruptor of human sleep, especially at particular times of day. Further dissection of this phenomenon necessitates modeling in a tractable model organism.

**Methods:**

Thus, we investigated the effects of light color on sleep in *Drosophila melanogaster*. We measured sleep in red-eyed Canton-S (CS) and white-eyed *w*^1118^ flies in baseline 12:12 light/dark conditions and experimental conditions with light-color (blue, red, or green) exposure for all 12 h of daylight or 3 h in the morning or evening.

**Results:**

Blue light reduced daytime and nighttime sleep in CS but not in *w*^1118^, potentially indicating a role for the compound eye in blue light’s effects on fruit fly sleep. Red light, especially in the evening, reduced sleep during exposure in both strains. Green light had minimal effects on sleep in CS flies, but evening exposure reduced sleep in *w*^1118^ flies, mimicking red light’s effects.

**Discussion:**

In conclusion, light’s effects on sleep in *D. melanogaster* are dependent on wavelength and time-of-day. Future studies will aim to dissect these mechanisms genetically.

## 1 Introduction

The study of life reached places as remote as Antarctica and the ocean floor long before researchers scientifically examined a phenomenon so universal it encompasses about one-third of our lives: sleep. As a physio-behavioral state, sleep is conserved across phyla from cnidarians to complex vertebrates (Kanaya et al., 2020; Nath et al., 2017), and overwhelming evidence supports nightly sleep’s importance for human health. Sleep disruption is a symptom of myriad neuro-psychiatric disorders (Breen et al., 2014; Jagannath et al., 2013; Ju et al., 2014; Wisor et al., 2005), and impaired sleep may play a role in the development of several common fatal diseases (Altman et al., 2012; Engeda et al., 2013; Medic et al., 2017). Hence, widespread sources of sleep disruption are serious public health concerns. With the omnipresence of modern technology, a new disruptor of sleep health has emerged: blue light-emitting visual display units, or screens.

The light emitted from screens on smartphones, TVs, and computers is enriched in blue wavelengths, which delay sleep onset and reduce both total and slow-wave sleep (Chang et al., 2015; Chellappa et al., 2013; Hale and Guan, 2015). Blue light’s sleep effects are time-of-day dependent, as evening exposure disrupts sleep that night and impairs wakefulness the following morning (Chang et al., 2015). The discovery of blue light’s deleterious impact on sleep has led to studies on how other light colors might affect sleep. Namely, exposure to red light may positively impact sleep health. For example, red light increases melatonin secretion and improves next-day wakefulness, as well as self-reported sleep quality scores (Kennedy et al., 2023; Zhao et al., 2012). In response to these discoveries, technology companies have developed software that mitigates negative sleep effects of blue light by enabling users to shift the spectral profile of their screens to longer wavelengths, but the efficacy of these technologies at improving sleep health remains unclear (Jin et al., 2021; Nagare et al., 2019). Clearly, light color and sleep have a complex relationship. However, we still lack a basic understanding of this important interaction, underscoring the need for a systematic investigation.

Studying sleep in human participants has immediate relevance to understanding broad phenomena related to human physiology, behavior, and disease; however, human subjects are unideal for dissecting the cellular and molecular pathways underlying sleep due to species-wide genetic and environmental variation. The fruit fly *Drosophila melanogaster* overcomes those barriers as an effective small model organism for studying environmental, genetic, and circuit-level control of complex behavior. Fly genetics are well-documented (Adams et al., 2000; Myers et al., 2000), and *D. melanogaster* has a fast generation time, allowing investigators to simultaneously monitor the sleep of large, inbred populations over a short timespan (Dissel, 2020). Unlike in humans, sleep in *D. melanogaster* has a bimodal pattern, with a daytime peak known as the “siesta” in addition to their nighttime sleep (see Figure 2B, e.g.). Nevertheless, sleep in flies shares core physiological and behavioral similarities with human sleep, such as: physical immobility, postural changes, lack of responsiveness, preferred location, dynamic stages, characteristic electrical patterns in the brain, and importantly, regulation by both circadian rhythms and a homeostatic response (Hendricks et al., 2000; Huber et al., 2004; Nitz et al., 2002; Shang et al., 2008; Shaw et al., 2000; Tainton-Heap et al., 2020; van Alphen et al., 2021; van Alphen et al., 2013).

Given these similarities, *D. melanogaster* has been employed as a model to study sleep behavior for decades (Hendricks et al., 2000; Shaw et al., 2002). However, to our knowledge, only one report has directly assayed the relationship between light color and sleep behavior in flies (Krittika and Yadav, 2022). Surprisingly, the authors found that blue light increased daytime sleep while red light moderately reduced daytime sleep, relative to white light. However, each individual fly was only exposed to one color of light throughout their adult lives, making it impossible to examine how animals adjust sleep behavior in response to environmental changes. Additionally, the study by Krittika and Yadav (2022) only utilized a full 12 h of light color exposure, providing little insight into how light color signaling differs across the day. While the effects of light color on fruit fly sleep remain relatively unknown, blue light shortens the overall lifespan of flies (Krittika and Yadav, 2022; Nash et al., 2019) and causes oxidative stress which leads to retinal degeneration, similar to humans (Chen et al., 2017; Nash et al., 2019; Shibuya et al., 2018). Coinciding with these negative health consequences, flies unsurprisingly possess an innate aversion to daytime blue light as well as a positive attraction to red light (Lazopulo et al., 2019). These phase-dependent color preferences are at least partially circadian clock-driven, and both the blue avoidance and red attraction behaviors peak midday when sunlight would typically be brightest (Lazopulo et al., 2019). Green light—which falls between blue and red light on the visual spectrum—may be beneficial to flies, as it extends their lifespan relative to blue or red light (Shen et al., 2021). Additionally, flies exhibit a clock- and compound eye-controlled preference for green light during the morning and evening hours (Lazopulo et al., 2019). Taken together, prior findings demonstrate that light color affects the physiology and behavior of *D. melanogaster* (likely in a time-dependent manner), but there remains a gap in the literature regarding how these three light colors—blue, red, and green light—specifically impact sleep/wake behavior in fruit flies across the day.

Therefore, we systematically tested how blue-, red-, and green-light exposure at different times of day affect sleep in *D. melanogaster*. In our study, we measured the sleep of two common *D. melanogaster* laboratory strains (Canton-S and *w*^1118^) in a baseline 12:12 white light/dark cycle before exposing them to one of the three light colors (blue, red, or green) during either all 12 h of the daytime, the first 3 h of daylight (“morning”), or the last 3 h of daylight (“evening”) for 6 experimental days. Here we provide a comprehensive, phenomenological portrait of how a core environmental stimulus—the color of light—regulates complex behavior in a tractable model organism.

## 2 Materials and methods

### 2.1 Fly husbandry

Two commonly used laboratory strains of *Drosophila melanogaster* were studied: the wild-type Canton-S (CS), as well as homozygous *white* mutants (*w*^1118^). *w*^1118^ animals were backcrossed 6 times onto a CS background to ensure genotypes had equivalent genetic backgrounds except for the *white* mutation. Homozygous female *w*^1118^ were identified phenotypically by their white eye color, and male *w*^1118^ with the mutation (carrying only one *white* allele) were identified similarly. All flies were raised at 25°C with a 12:12 white light/dark (LD) photoperiod on cornmeal/yeast food as previously described (Juneau et al., 2019). Young adult flies were ≥ 3 d post-eclosion at the start of the experiment, as adult sleep behavior stabilizes by day 3 (Shaw et al., 2000). Within a given study, all flies had eclosed within 4 days of one another. Female flies were mated with males for at least 2 days prior to experimentation to ensure that no virgin females, which have unique sleep patterns (Dove et al., 2017; Isaac et al., 2010), were included in the female sleep data.

### 2.2 Sleep testing

After allowing them to age and mate, flies were anesthetized with CO_2_ and carefully loaded into polycarbonate tubes (5 mm diameter × 65 mm length) with food (5% sucrose w/v, 2% agar w/v). Tubes were then placed into *Drosophila* Activity Monitor (DAM2) boards (Trikinetics, Inc., Waltham, MA, USA). Infrared beams within the DAM2 boards record each time a fly crosses the center of tube. All activity monitors, along with an environmental monitor, were loaded into the same incubator. The incubator maintained an internal environment of ∼25.0°C and ∼50–75% humidity during the sleep studies. For each experiment, monitors were loaded into the incubator the day before experimentation began. The activity monitors recorded each fly’s activity for two 12 h:12 h white light/dark (WD) baseline days, followed by 6 experimental days of light color treatment, and then 2 WD recovery days.

### 2.3 Light treatment

**FIGURE 1:**
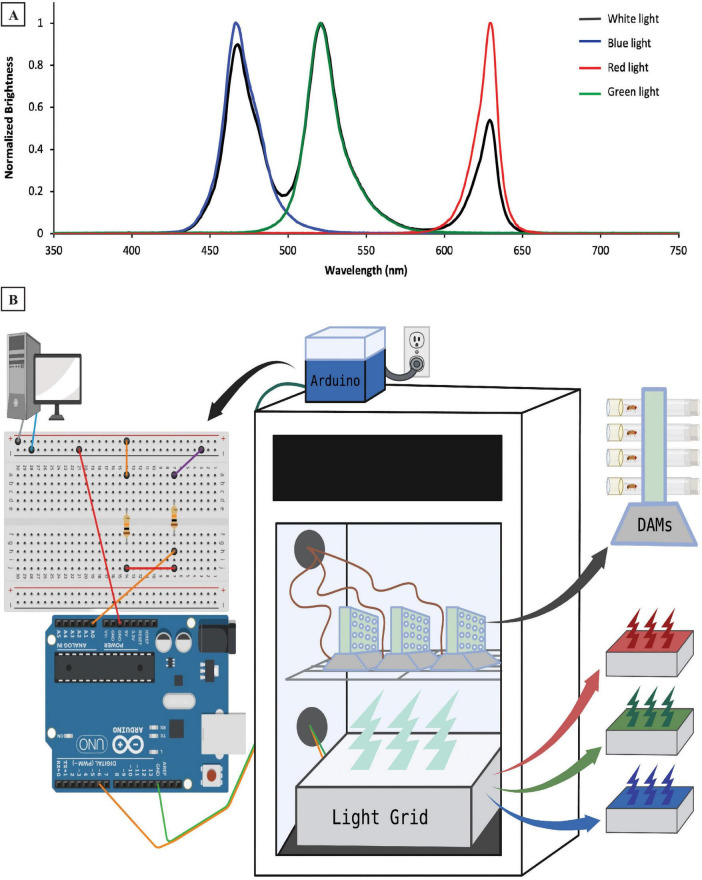
Experimental design and methodology. **(A)** Emission spectra of four light colors (white, blue, red, and green) used during the experiment. **(B)** Experimental setup illustrating locations of hardware controlling the light emission, as well as the *Drosophila* Activity Monitors (DAMs) in and around our sleep testing incubator. The desktop computer sends electrical signals to a circuit that then connects to our Arduino microcontroller (atop the incubator), which is wired to the light grid (inside the incubator). The light grid—which contains red, green, and blue LEDs, is located below the DAMs, which track individual flies’ sleep/wake behavior. The DAMs are wired out of the incubator to provide data to the desktop computer.

On the 6 experimental days of each experiment, flies were illuminated with either blue light (peak λ = 466 nm), green light (peak λ = 521 nm), or red light (peak λ = 629 nm) during their subjective daytime (Figure 1A). Zeitgeber time (ZT) 0 was set to be the onset of light each day. Light color exposure was studied during all 12 h of the daytime (ZT0–ZT12), the first 3 h of the day (ZT0–ZT3), and the last 3 h before the dark period (ZT9–ZT12). For the 3 h-exposure experiments, flies were illuminated with white light (3 peaks at 468, 521, and 629 nm) during the other 9 h of the light period. All spectral measurements were taken using a Red Tide USB650 spectrometer (Ocean Insight, Inc., Orlando, FL, USA). White, blue, green, and red light over all 10 d of each experiment were emitted from the same source: a homemade RGB-emitting light box located directly below the activity monitors (Figure 1B). The RGB light box contained three LEDs per bulb (one red, one green, and one blue), as well as a white diffuser sheet placed over the LEDs. The homemade light box’s color changes were controlled with an Arduino microcontroller (Arduino LLC, Boston, MA, USA), which in turn, received time-of-day signals via electrical pulses from the DAM System (Figure 1B). A one-second electrical pulse was sent to the Arduino every 12 h to maintain the 12:12 LD photoperiod in all experiments. Additionally, the 3-h exposure experiments shown in Figures 3, 5, 7 also had a one-second electrical pulse every 24 h to shift between white light and colored light during the day for 3 h. These electrical signals initiated transitions between steps in the lighting paradigms established in Arduino scripts (“sketches”) designed in our lab that also dictated the color and intensity of the light to be delivered. Intensities ranged from ∼250–300 lx for blue and green light, whereas the red LED had a lower intensity (∼150 lx). By adjusting the electrical output strength in the Arduino sketch, we lowered the LED emission intensity during the white-light condition until the overall light intensity in the incubator was ∼230 lx, approximating levels seen with the individual light colors.

### 2.4 Data analysis

Sleep and circadian data were extracted from the activity dataset with the Sleep and Circadian Analysis MATLAB Program (SCAMP), versions 2–4 (Donelson et al., 2012; Vecsey et al., 2024). Sleep was defined as ≥ 5 min of inactivity (no beam crosses), as is widely done in the field (Hendricks et al., 2000). Data were only used from flies that survived the entirety of the experiment.

**FIGURE 2:**
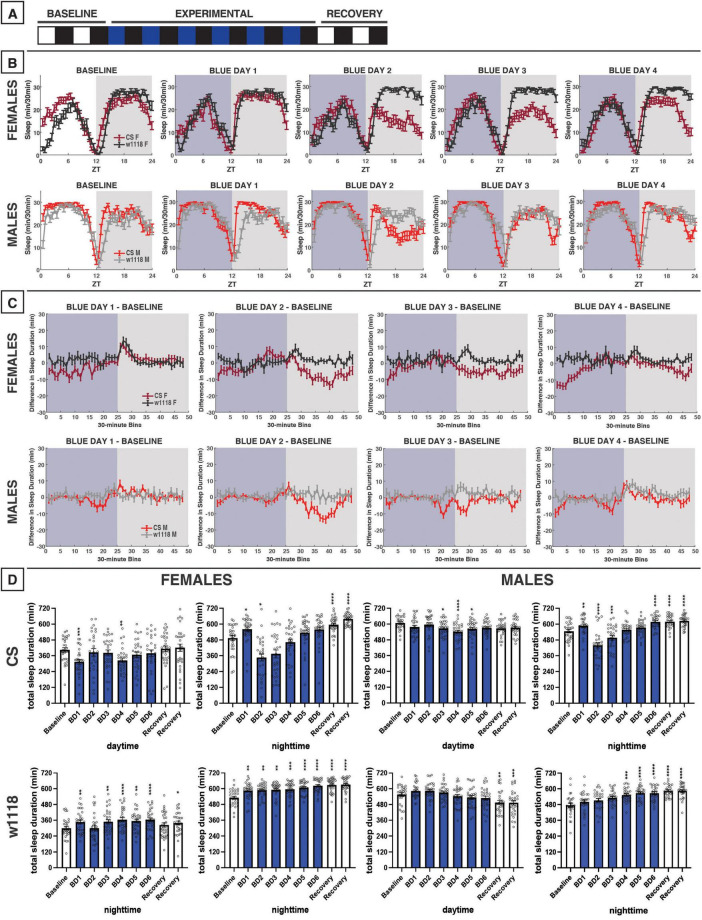
Blue-light exposure for 12 h reduces daytime and nighttime sleep in male and female Canton-S (CS) *D. melanogaster*, but not *white* (*w*^1118^) mutants. **(A)** Timeline of entire experiment, showing 2 d of baseline 12:12 white/dark (WD), 6 d experimental 12:12 blue/dark (BD), and 2 d recovery WD. **(B)** Sleep patterns for male and female CS (red) and *w*^1118^ (gray) flies. Sleep (min) during each 30-min bin of the day was averaged across flies for each group and plotted to show overall sleep profiles over the second baseline and first four experimental days. **(C)** Differences in sleep between experimental days and baseline of male and female CS and *w*^1118^. Mean sleep duration (min) in each 30-min bin of the baseline day was subtracted from the mean sleep duration in each respective bin on the first four experimental days. **(D)** Total sleep duration (min) during day (ZT0–12) and night (ZT12–24) for all groups. All experimental and recovery days of the experiment are shown, along with the second baseline day. Sexes and genotypes were analyzed separately with a repeated-measures ANOVA or nonparametric alternative, followed by *post-hoc* tests. Asterisks indicates significant difference in day/night sleep (per *post-hoc* test) within group between experimental/recovery and baseline day (*P < 0.05, **P < 0.01, ***P < 0.001, ****P < 0.0001). Ns were 30, 31, 29, 29 for CS female, *w*^1118^ female, CS male, *w*^1118^ male, respectively.

**FIGURE 3:**
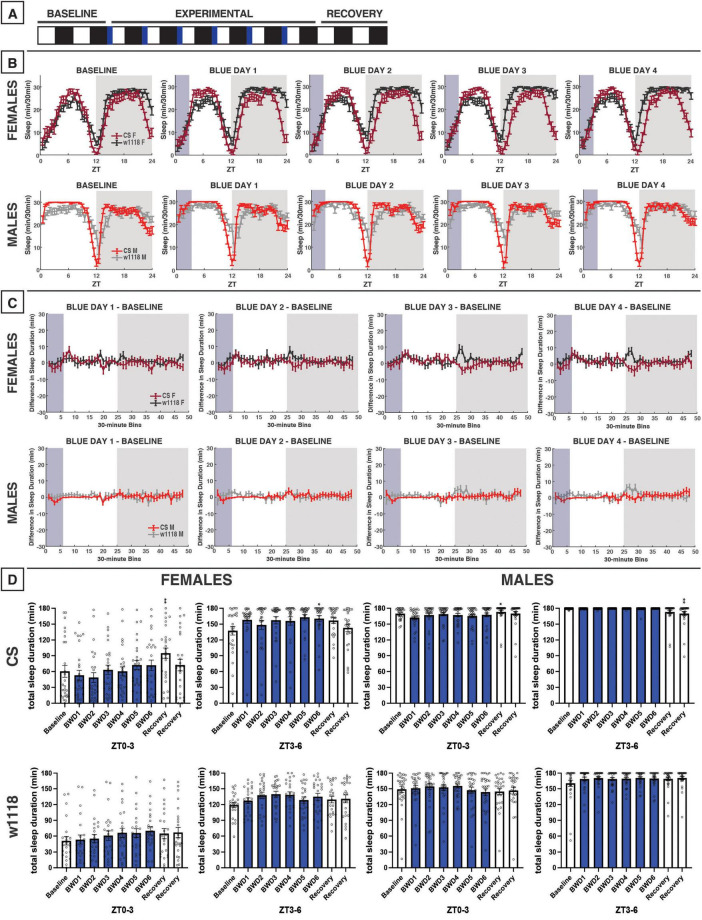
Morning blue light for 3 h marginally impacts sleep in CS or *w*^1118^ flies. **(A)** Timeline of entire experiment, showing 2 d of baseline 12:12 WD, 6 d experimental 3:9:12 blue/white/dark (BWD), 2 d recovery WD. **(B)** Sleep patterns for male and female CS (red) and *w*^1118^ (gray) flies. Sleep (min) during each 30-min bin of the day was averaged for across flies for each group and plotted to show the overall sleep profiles over the second baseline and first four experimental days. **(C)** Differences in sleep between experimental days and baseline of male and female CS and *w*^1118^. Mean sleep duration (min) in each 30-min bin of the baseline day was subtracted from the mean sleep duration in each respective bin on the first four experimental days. **(D)** Total sleep duration (min) during blue light exposure (ZT0–3) and immediately after (ZT3–6). Sexes and genotypes were analyzed separately with a repeated-measures ANOVA or nonparametric alternative, followed by *post-hoc* tests. Asterisks indicates significant difference in sleep during 3-h bin (per *post-hoc* test) within group between experimental/recovery and baseline day (*P < 0.05, **P < 0.01). Ns were 27, 24, 29, 29 for CS female, *w*^1118^ female, CS male, *w*^1118^ male, respectively.

**FIGURE 4:**
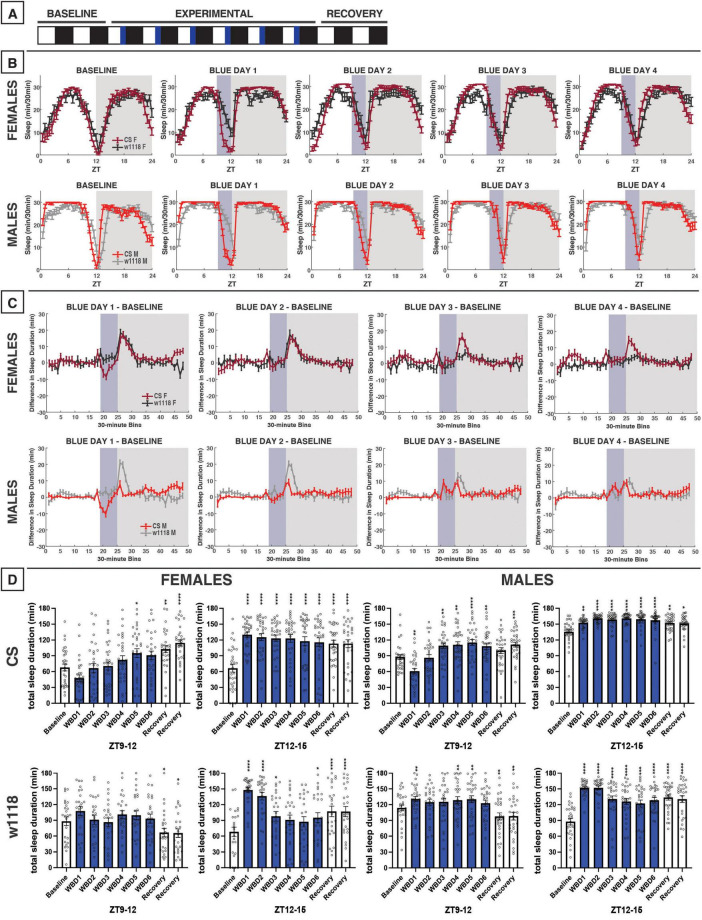
Evening blue light for 3 h acutely reduces sleep in CS flies and increases nighttime sleep both CS and *w*^1118^. **(A)** Timeline of entire experiment, showing 2 d of baseline 12:12 WD, 6 d experimental 9:3:12 white/blue/dark (WBD), 2 d recovery WD. **(B)** Sleep patterns for male and female CS (red) and *w*^1118^ (gray) flies. Sleep (min) during each 30-min bin of the day was averaged across flies for each group and plotted to show overall sleep profiles over the second baseline and first four experimental days. **(C)** Differences in sleep between experimental days and baseline of male and female CS and *w*^1118^. Mean sleep duration (min) in each 30-min bin of the baseline day was subtracted from the mean sleep duration in each respective bin on the first four experimental days. **(D)** Total sleep duration (min) during the 3-h blue light exposure (ZT9–12) and the 3 h immediately after (ZT12–15). Sexes and genotypes were analyzed separately with a repeated-measures ANOVA or nonparametric alternative, followed by *post-hoc* tests. Asterisks indicates significant difference in sleep during 3-h bin (per *post-hoc* test) within group between experimental/recovery and baseline day (*P < 0.05, **P < 0.01, ***P < 0.001, ****P < 0.0001). Ns were 31, 25, 29, 29 for CS female, *w*^1118^ female, CS male, *w*^1118^ male, respectively.

**FIGURE 5:**
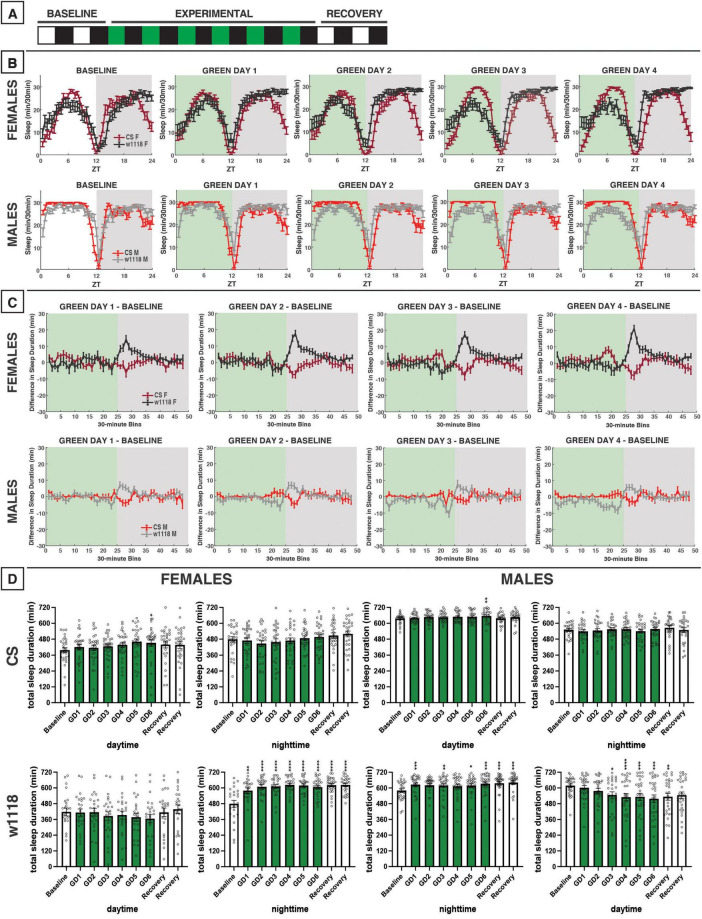
Green-light exposure for 12 h does not alter sleep in CS flies but does in *w*^1118^. **(A)** Timeline of entire experiment, showing 2 d of baseline 12:12 white/dark (WD), 6 d experimental 12:12 green/dark (GD), 2 d recovery WD. **(B)** Sleep patterns for male and female CS (red) and *w*^1118^ (gray) flies. Sleep (min) during each 30-min bin of the day was averaged across flies for each group and plotted to show overall sleep profiles over the second baseline and first four experimental days. **(C)** Differences in sleep between experimental days and baseline of male and female CS and *w*^1118^. Mean sleep duration (min) in each 30-min bin of the baseline day was subtracted from the mean sleep duration in each respective bin on the first four experimental days. **(D)** Total sleep duration (min) during day (ZT0–12) and night (ZT12–24) for all groups. All experimental and recovery days of the experiment are shown, along with the second baseline day. Sexes and genotypes were analyzed separately with a repeated-measures ANOVA or nonparametric alternative, followed by *post-hoc* tests. Asterisks indicates significant difference in day/night sleep (per *post-hoc* test) within group between experimental/recovery and baseline day (*P < 0.05, **P < 0.01, ***P < 0.001, ****P < 0.0001). Ns were 29, 26, 27, 29 for CS female, *w*^1118^ female, CS male, *w*^1118^ male, respectively.

**FIGURE 6:**
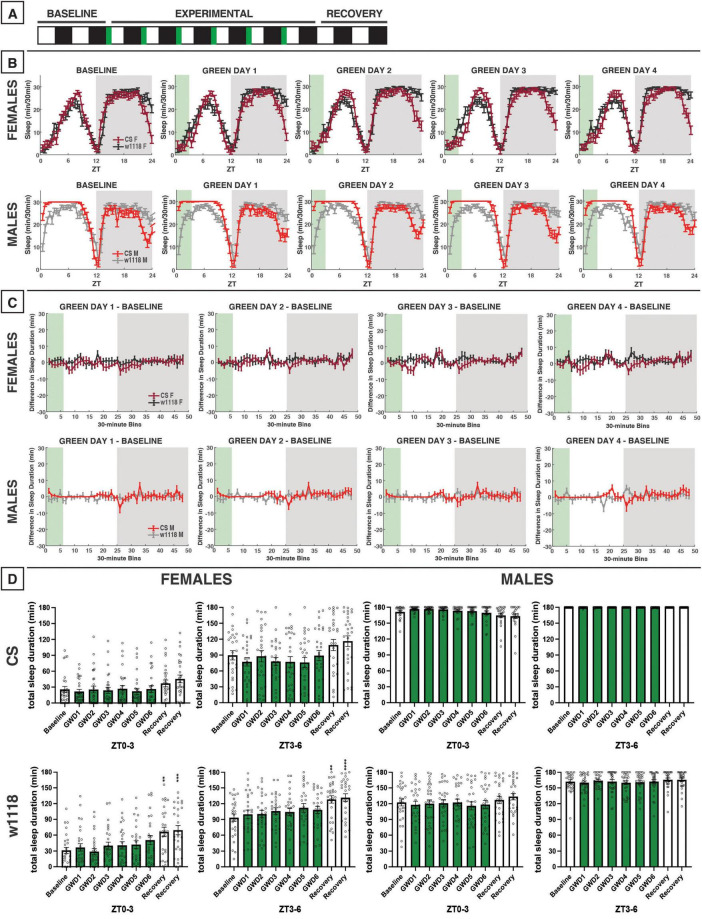
Morning green light for 3 h has little effect on sleep in CS and *w*^1118^ flies. **(A)** Timeline of entire experiment, showing 2 d of baseline 12:12 WD, 6 d experimental 3:9:12 green/white/dark (GWD), 2 d recovery WD. **(B)** Sleep patterns for male and female CS (red) and *w*^1118^ (gray) flies. Sleep (min) during each 30-min bin of the day was averaged for all groups and plotted to show the flies’ overall sleep profiles over the second baseline and first four experimental days. **(C)** Difference in sleep between experimental days and baseline of male and female CS and *w*^1118^. Mean sleep duration (min) in each 30-min bin of the baseline day was subtracted from the mean sleep duration in each respective bin on the first four experimental days. **(D)** Total sleep duration (min) during green-light exposure (ZT0–3) and the 3 h preceding exposure after (ZT21–24). Sexes and genotypes were analyzed separately with a repeated-measures ANOVA or nonparametric alternative, followed by *post-hoc* tests. Asterisks indicates significant difference in sleep during 3-h bin (per *post-hoc* test) within group between experimental/recovery and baseline day (**P < 0.01, ***P < 0.001, ****P < 0.0001). Ns were 29, 27, 21, 29 for CS female, *w*^1118^ female, CS male, *w*^1118^ male, respectively.

**FIGURE 7:**
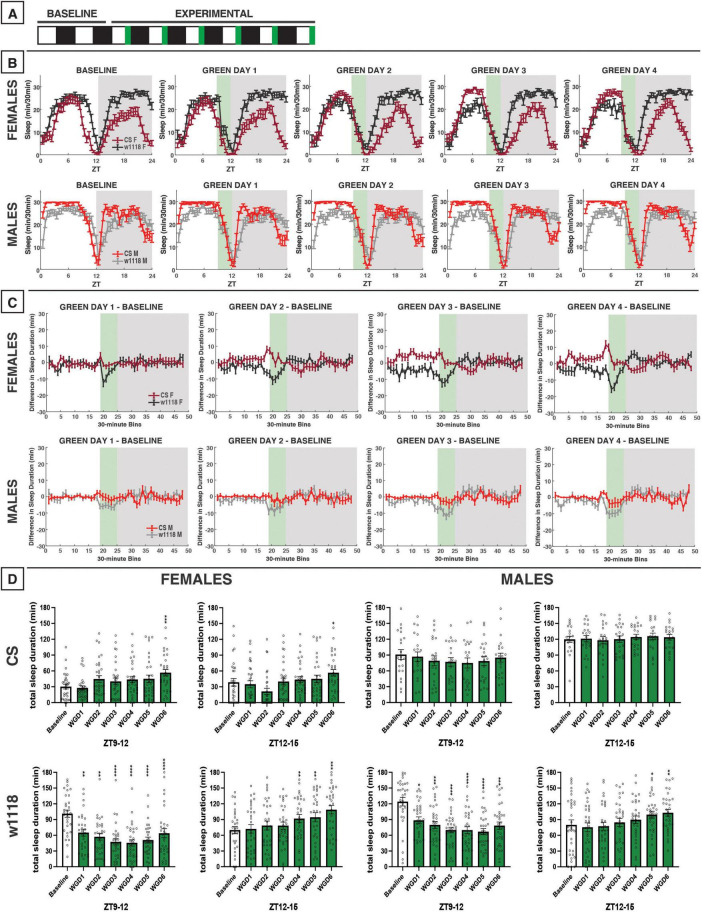
Evening green light for 3 h acutely reduces sleep in male and female *w*^1118^ but not in CS flies. **(A)** Timeline of entire experiment, showing 2 d of baseline 12:12 WD, 6 d experimental 9:3:12 white/green/dark (WGD). **(B)** Sleep patterns for male and female CS (red) and *w*^1118^ (gray) flies. Sleep (min) during each 30-min bin of the day was averaged across flies for each group and plotted to show overall sleep profiles over the second baseline and first four experimental days. **(C)** Differences in sleep between experimental days and baseline of male and female CS and *w*^1118^. Mean sleep duration (min) in each 30-min bin of the baseline day was subtracted from the mean sleep duration in each respective bin on the first four experimental days. **(D)** Total sleep duration (min) during green-light exposure (ZT9–12) and immediately after (ZT12–15). Sexes and genotypes were analyzed separately with a repeated-measures ANOVA or nonparametric alternative, followed by *post-hoc* tests. Asterisks indicates significant difference in sleep during 3-h bin (per *post-hoc* test) within group between experimental/recovery and baseline day (*P < 0.05, **P < 0.01, ***P < 0.001, ****P < 0.0001). Ns were 31, 31, 21, 32 for CS female, *w*^1118^ female, CS male, *w*^1118^ male, respectively.

**FIGURE 8:**
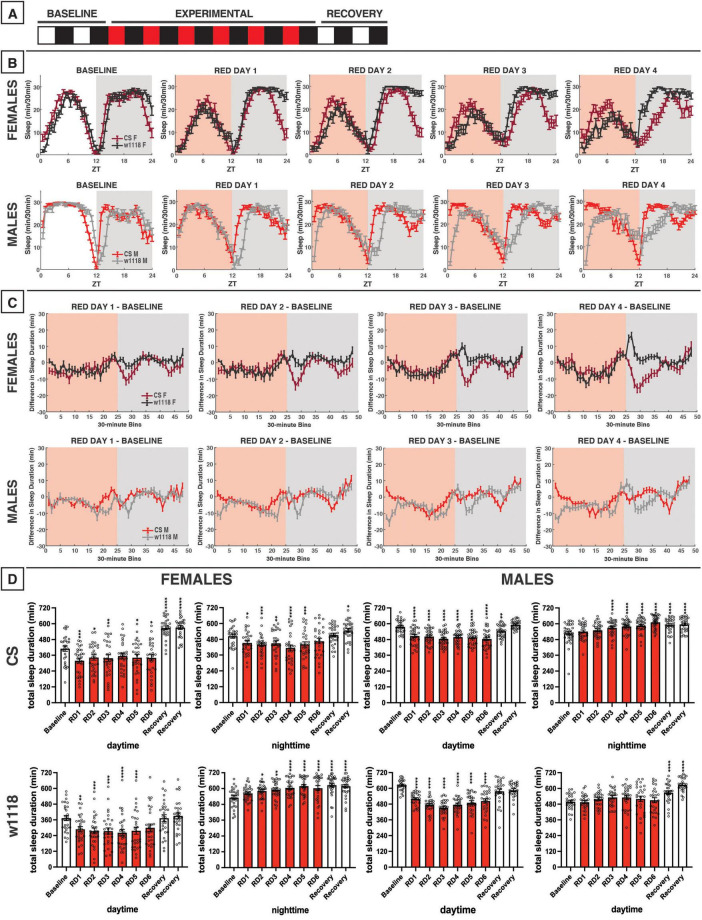
Red-light exposure for 12 h acutely reduces daytime sleep and remodels overall sleep patterns in CS and *w*^1118^. **(A)** Timeline of entire experiment, showing 2 d of baseline 12:12 white/dark (WD), 6 d experimental 12:12 red/dark (RD), 2 d recovery WD. **(B)** Sleep patterns for male and female CS (red) and *w*^1118^ (gray) flies. Sleep (min) during each 30-min bin of the day was averaged across flies for each group and plotted to show overall sleep profiles over the second baseline and first four experimental days. **(C)** Differences in sleep between experimental days and baseline of male and female CS and *w*^1118^. Mean sleep duration (min) in each 30-min bin of the baseline day was subtracted from the mean sleep duration in each respective bin on the first four experimental days. **(D)** Total sleep duration (min) during day (ZT0–12) and night (ZT12–24) for all groups. All experimental and recovery days of the experiment are shown, along with the second baseline day. Sexes and genotypes were analyzed separately with a repeated-measures ANOVA or nonparametric alternative, followed by *post-hoc* tests. Asterisks indicates significant difference in day/night sleep (per *post-hoc* test) within group between experimental/recovery and baseline day (*P < 0.05, **P < 0.01, ***P < 0.001, ****P < 0.0001). Ns were 29, 29, 32, 27 for CS female, *w*^1118^ female, CS male, *w*^1118^ male, respectively.

**FIGURE 9:**
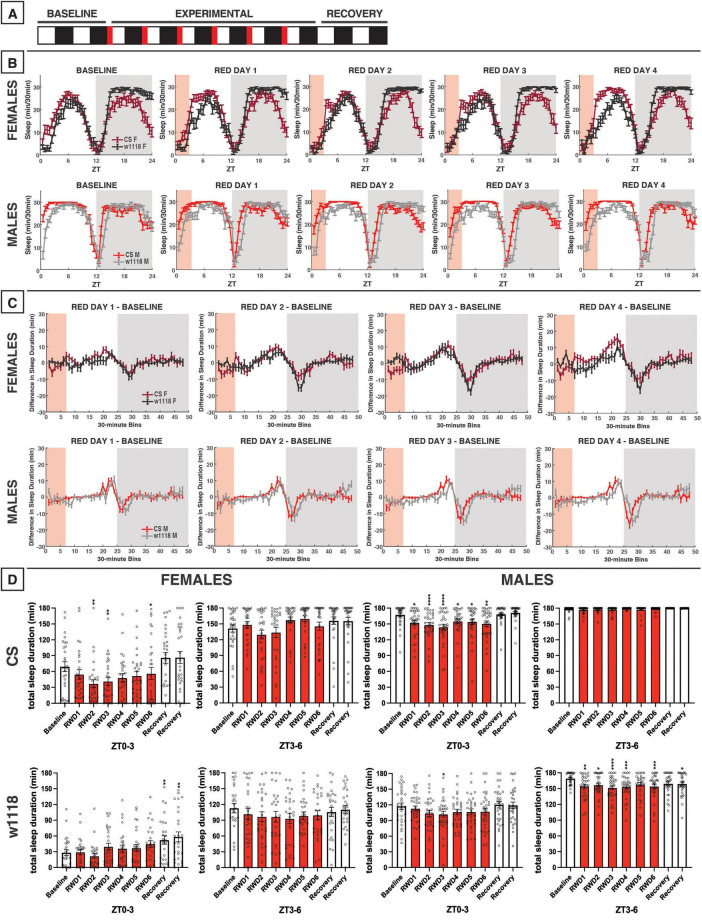
Morning red light extends the siesta and delays nighttime sleep in CS and *w*^1118^. **(A)** Timeline of entire experiment, showing 2 d of baseline 12:12 WD, 6 d experimental 3:9:12 red/white/dark (RWD), 2 d recovery WD. **(B)** Sleep patterns for male and female CS (red) and *w*^1118^ (gray) flies. Sleep (min) during each 30-min bin of the day was averaged across flies for each group and plotted to show overall sleep profiles over the second baseline and first four experimental days. **(C)** Differences in sleep between experimental days and baseline of male and female CS and *w*^1118^. Mean sleep duration (min) in each 30-min bin of the baseline day was subtracted from the mean sleep duration in each respective bin on the first four experimental days. **(D)** Total sleep duration (min) during red-light exposure (ZT0–3) and during the last 3 h of daytime (ZT9–12). Sexes and genotypes were analyzed separately with a repeated-measures ANOVA or nonparametric alternative, followed by *post-hoc* tests. Asterisks indicates significant difference in sleep during 3-h bin (per *post-hoc* test) within group between experimental/recovery and baseline day (*P < 0.05, **P < 0.01, ***P < 0.001, ****P < 0.0001). Ns were 29, 27, 32, 31 for CS female, *w*^1118^ female, CS male, *w*^1118^ male, respectively.

**FIGURE 10:**
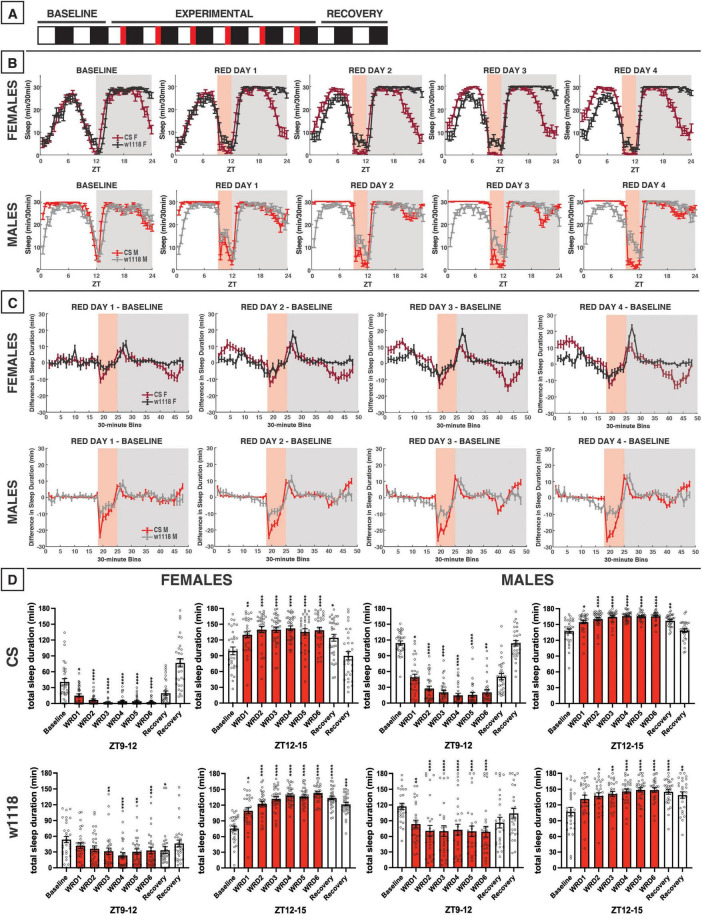
Evening red light for 3 h acutely reduces sleep in both CS and *w*^1118^ flies, but stronger effects are observed in CS. **(A)** Timeline of entire experiment, showing 2 d of baseline 12:12 WD, 6 d experimental 9:3:12 white/red/dark (WRD), 2 d recovery WD. **(B)** Sleep patterns for male and female CS (red) and *w*^1118^ (gray) flies. Sleep (min) during each 30-min bin of the day was averaged across flies for each group and plotted to show overall sleep profiles over the second baseline and first four experimental days. **(C)** Differences in sleep between experimental days and baseline of male and female CS and *w*^1118^. Mean sleep duration (min) in each 30-min bin of the baseline day was subtracted from the mean sleep duration in each respective bin on the first four experimental days. **(D)** Total sleep duration (min) during red-light exposure (ZT9–12) and immediately after (ZT12–15). Sexes and genotypes were analyzed separately with a repeated-measures ANOVA or nonparametric alternative, followed by *post-hoc* tests. Asterisks indicates significant difference in sleep during 3-h bin (per *post-hoc* test) within group between experimental/recovery and baseline day (*P < 0.05, **P < 0.01, ***P < 0.001, ****P < 0.0001). Ns were 32, 31, 30, 28 for CS female, *w*^1118^ female, CS male, *w*^1118^ male, respectively.

Each 24-h day’s sleep/activity data were divided into 12-h, 3-h, and 30-min bins to enable both broad day/night sleep analysis (12-h bins) and finer-tuned dissection of sleep patterns. Next, total minutes of sleep were calculated for each bin on baseline and experimental days. Each group’s binned data across days were subjected to Kolmogorov–Smirnov and Shapiro–Wilk normality tests. For normally distributed data, we carried out a within-group, repeated-measures ANOVA to analyze how sleep changed across days. For non-Gaussian data, a nonparametric equivalent of repeated-measures ANOVA (Friedman’s test) was performed. If a significant effect was identified via ANOVA or Friedman’s test, we performed *post*-*hoc* Tukey or Dunn tests, respectively, to ascertain within a particular group which experimental/recovery days differed significantly from baseline sleep. A *p*-value < 0.05 was considered significant for all statistical tests. Significant results for *post-hoc* tests are indicated with asterisks (*) in panel D of Figures 2–10, while ANOVA and Friedman’s test results are reported in Supplementary Tables 1, 2 respectively. Statistical analysis was carried out using GraphPad Prism 10 (Dotmatics, Boston, MA, USA).

Because experiments were performed with either 12-h (Figures 2, 5, 8) or 3-h light-color exposures (Figures 3, 4, 6, 7, 9, 10), data were analyzed and displayed accordingly (see panel D for each figure). For instance, data in 12-h bins (day and night) are shown and discussed for 12-h light exposures (e.g., Figure 2), while data in 3-h bins (ZT0–3, ZT3–6, etc.) are shown for 3-h exposure experiments to demonstrate acute effects during (and immediately after) the light color exposure (see panel D for Figures 3, 4, 6, 7, 9, 10).

## 3 Results

### 3.1 12 h of blue-light exposure

To investigate the effects of blue-light exposure on *D. melanogaster* sleep, we measured the baseline sleep of CS and *w*^1118^ flies in 12:12 white/dark (WD) for 2 days before transitioning to 12:12 blue/dark (BD) for 6 days and then back to WD for 2 days of recovery (Figure 2A). As sleep is sexually dimorphic in flies, the two sexes were analyzed separately. For this experiment and all others, daytime sleep effects for each sex and genotype will be discussed prior to nighttime sleep effects.

Female CS flies tended to sleep less during the daytime under blue light exposure compared to the baseline day (Figures 2B–D). In contrast, no acute reductions in sleep during the blue-light exposure were observed in *w*^1118^ females (Figures 2B–D). Like females, male CS flies, but not *w*^1118^, exhibited substantial daytime sleep effects due to 12 h of blue light (Figures 2B–D). Notably, the sleep-reducing effect of 12-h blue-light exposure on CS females and males was primarily observed during the morning hours (Figures 2B, C).

We had expected that blue light exposure would delay sleep onset. However, CS and *w*^1118^ flies surprisingly showed significant increases in nighttime sleep between baseline and the first day of blue/dark treatment (BD1) (Figures 2B–D). Substantial nighttime sleep loss began to emerge on BD2, as both male and female CS flies reduced their nighttime sleep by > 25% (Figures 2B–D). Over subsequent days, nighttime sleep gradually returned back to baseline levels. In contrast, *w*^1118^ flies of both sexes did not show any reduction in nighttime sleep on BD2, and instead generally slept *more* at night following blue-light exposure than during the baseline day (Figure 2D). Taken together, these findings indicate a potent daytime- and nighttime-sleep-inhibiting effect of blue light that is present in CS flies but not *w*^1118^ flies.

### 3.2 3 h of morning blue light

We next sought to examine how 3 h of blue-light exposure during the flies’ subjective morning or evening alters their sleep (Figures 3, 4). These 3-h experiments allowed us to test for time-specific effects of blue light on sleep, potentially to better model human screen use when waking up vs. before bed.

Unlike the 12-h exposure, blue light restricted to 3 h in the morning did not globally alter CS sleep patterns in an overt manner (Figure 3B). Accordingly, limited effects were observed on total sleep within 3-h bins in females nor males, including the 3-h light exposure (ZT0–3) and the following 3-h bin (ZT3–6) (Figure 3D), suggesting that blue light’s sleep effects on flies may depend on what time of day the exposure occurs.

### 3.3 3 h of evening blue light

After testing the effects of morning blue light, we sought to examine how evening exposure to blue light affected *D. melanogaster* sleep patterns (Figure 4). On the first day of evening blue light exposure, both male and female CS flies had reduced sleep during the 3 h of blue-light exposure, compared to baseline sleep across that same 3-h period (ZT9–12), although this only reached significance in males (Figures 4B–D). This sleep reduction was not observed in either male or female CS over subsequent blue-light exposures, suggesting that flies acclimated to the transition to blue light. In fact, males began to sleep *more* during the blue light exposure over successive days. In contrast to CS flies, sleep in *w*^1118^ males and females was increased from baseline across blue-light treatment days, including from the first exposure (Figures 4B–D).

We expected that evening exposure to blue light—close to the onset of nighttime sleep—would decrease CS nighttime sleep as was observed in response to 12 h of blue light exposure. However, our results indicated the opposite. Blue light induced an increase in nighttime sleep for about 3 h following the onset of darkness in both CS and *w*^1118^ females (Figures 4B, C). In male flies, this effect was stronger in *w*^1118^ than CS (Figure 4C), but this greater magnitude appeared to stem from *w*^1118^ males having a longer sleep latency (or later sleep onset) at night than CS males (Figure 4B). Overall, evening exposure to blue light caused an acute sleep reduction during the exposure that was specific to CS flies and subsided over successive days, and caused increased sleep in the first portion of the dark period in both genotypes that persisted throughout the experimental days.

### 3.4 12 h of green-light exposure

We next investigated the effects of green light on *D. melanogaster* sleep using a 12:12 green/dark (GD) schedule (Figure 5A). Green light elicited little change in daytime sleep in male and female flies in both CS and *w*^1118^ strains (Figures 5B–D). As with daytime sleep, no changes in nighttime sleep in CS males or females were observed due to the 12-h green-light exposure (Figures 5B–D). However, *w*^1118^ males and females had an increase in sleep early in the night due to an earlier sleep onset (Figures 5B–D). Overall, switching from white-light to green-light had less of an effect on sleep than switching to blue-light exposure.

### 3.5 3 h of morning green light

To study the effects of morning green-light exposure on *D. melanogaster* sleep, we used the same experimental design as our morning blue-light experiment but with a 3:9:12 green/white/dark (GWD) photoperiod during the experimental phase (Figure 6A). This morning exposure to green light produced little change in daytime or nighttime sleep of male and female CS and *w*^1118^ flies (Figures 6B–D). These results were very similar to what we observed in response to morning exposure to blue light (Figure 3).

### 3.6 3 h of evening green light

We next performed an evening green-light exposure experiment, as we did with blue light. During the experimental period, flies were housed on a photoperiod of 9:3:12 WGD; however, no recovery data in WD were able to be obtained (Figure 7A). Because 12 h of green light had little effect on daytime sleep in CS females (Figure 5), we anticipated similar results with the evening exposure. Indeed, no significant effects of the evening green-light exposure were observed in CS males or females (Figures 7B–D). However, we found that *w*^1118^ males and females were acutely sensitive to evening green light, with sharp reductions in sleep occurring during the 3-h exposure from ZT9–12 (Figures 7B–D).

Nighttime sleep was not substantially altered in either CS or *w*^1118^ flies. This was notably different from the response to 12 h of green-light exposure, which had resulted in an increase in sleep in *w*^1118^ flies in the early nighttime.

### 3.7 12 h of red-light exposure

We next investigated the effects of red light on *D. melanogaster* sleep using a 12:12 red/dark (RD) schedule (Figure 8A). In CS and *w*^1118^ female flies under red-light conditions, the siesta peaked earlier in the day as compared with baseline white-light conditions (Figure 8B). In addition to this change in pattern, total daytime sleep was significantly reduced in both CS and *w*^1118^ females across most experimental days (Figure 8D). After returning to a WD photoperiod in the recovery phase of the experiment, CS flies sharply increased their daytime sleep, far surpassing their baseline levels (Figure 8D)—a possible rebound effect.

Male CS flies also had changes in daytime sleep under red-light conditions that were even more obvious than in female flies. Their siesta was strongly phase-advanced, resulting in the loss of their morning arousal period and an earlier start to their evening arousal period (Figure 8B). Interestingly, male *w*^1118^ flies exhibited an opposite effect on morning sleep as compared to CS flies, having stronger morning arousal as the experiment progressed (Figures 8B, C). The end result of these observed potent sleep effects was that both CS and *w*^1118^ male flies had significantly reduced daytime sleep on all 6 red-light days (Figure 8D). Male and female *w*^1118^ flies also began to lose their period of wakefulness at dusk across days of red-light exposure (Figure 8B).

Nighttime sleep effects of red light were less conclusive and consistent than those observed in the daytime. In females, the two genotypes showed opposing effects. For instance, *w*^1118^ flies progressively increased their total nighttime sleep, reaching peak levels on RD5 (Figure 8D). This increased total nighttime sleep was driven mainly by increases early in the night (Figures 8B, C). On the contrary, CS females showed a delay in their nighttime sleep onset during the RD days (Figure 8B). With the return the WD, CS females recovered to their baseline nighttime sleep amount (Figure 8D).

The most notable change to nighttime sleep in CS males was that sleep time gradually increased across red-light exposure days. This seemed to be driven by a phase advance in which flies experienced a mild morning arousal at approximately ZT21, followed by an increase in sleep that continued through “lights-on” at ZT0 (Figure 8B). *w*^1118^ males showed no significant changes relative to baseline in their total nighttime sleep during the red-light days (Figure 8D); however, their sleep patterns changed dramatically (Figure 8B). *w*^1118^ males gradually began to show a more gradual sleep onset, achieving peak nighttime sleep levels much later during the RD days than during the WD baseline (Figures 8B, C). Overall, these data demonstrate that red light has a genotype- and sex-independent, inhibitory effect on daytime sleep but genotype-specific nighttime sleep effects. The timing of sleep/activity is also affected by red-light exposure, with red light causing a phase advance in daytime sleep in all groups, but especially males, and delays in sleep onset in male *w*^1118^ flies.

### 3.8 3 h of morning red light

We next tested the effects of morning red-light exposure using a 3:9:12 red/white/dark (RWD) photoperiod during the six experimental days (Figure 9A). The results demonstrated that morning red-light exposure elicited only minor effects during the exposure, but, in females in particular, caused an immediate increase in sleep as soon as the light condition was switched from red-light to white-light—this is most noticeable on RWD days 2–4 (Figure 9B). In RWD conditions, both CS females and males also experienced an extension of the siesta into the afternoon/evening, and then a delay in sleep onset after lights-out (Figures 9B, C), which was quite different from the response to 12 h of red-light exposure (Figures 8B, C). This phase delay resulted in an increase in sleep toward the end of the light period and a decrease in sleep in the beginning of the dark period (Figure 9C). This overall pattern was also observed in both female and male *w*^1118^ flies. Taken together, these results show that a 3 h morning red-light pulse has little acute effects on sleep but may remodel both CS and *w*^1118^ sleep patterns by delaying their sleep/activity timing later in the day.

### 3.9 3 h of evening red light

During our 12-h experiment with red light, flies showed substantial reductions in daytime sleep late during the siesta (Figure 8). Thus, we expected that exposing flies to evening red light using a 9:3:12 white/red/dark photoperiod (WRD) (Figure 10A) would elicit strong sleep reductions during the evening red-light exposure. Our results matched this expectation, as both female and male CS flies exhibited potent sleep reductions during the WRD portion of the experiment, especially at the onset of red light at ZT9 (Figures 10B–D). CS females and males also had increased sleep in the morning, an effect that increased over successive WRD days (Figure 10C). While *w*^1118^ flies followed qualitatively similar patterns to those observed in CS flies, they were not as completely aroused by the onset of the evening red-light period (Figures 10B–D).

In terms of the effects of evening red-light exposure on nighttime sleep patterns, both *w*^1118^ and CS females and males had increased sleep soon after the onset of darkness (Figure 10C). Although *w*^1118^ showed a weaker sleep reduction *during* the red-light exposure, this genotype exhibited a robust nighttime sleep increase immediately *after* red-light offset (Figure 10C). Indeed, all groups showed significant increases in sleep in the ZT12–15 period during WRD days (Figure 10D). Despite similar effects of evening red light on sleep early in the night, the two genotypes diverged during the latter portion of the night. As the experiment progressed, CS females began to have reduced nighttime sleep (ZT18–24), while *w*^1118^ females did not deviate from their baseline late-night sleep pattern (Figures 10B, C). In the experiment involving 12 h of red light exposure, CS males had appeared to phase-advance their morning anticipation activity bout (Figure 8B), and a similar effect was noted with the 3-h evening exposure (Figure 10B). *w*^1118^ males, on the other hand, exhibited little change to their sleep late at night after the evening red-light exposure (Figure 10C).

It is notable, especially in CS females, that evening red-light exposure tended to increase sleep at both the beginning of the day and beginning of the night, while decreasing sleep toward the end of the day and the end of the night (Figure 10C). In contrast, morning red-light exposure had the opposite effect (Figure 9C). These changes seem to stem from a phase advance due to evening red-light exposure and a phase-delay due to morning red-light exposure. Additionally, the responses of *w*^1118^ flies to red light were generally more similar to CS flies’ than their responses to other colors, although there were still some differences between the two genotypes.

## 4 Discussion

### 4.1 Sleep-suppressing effects of blue light in wild-type flies

Blue light appears to act as an alerting stimulus in humans (Chang et al., 2015), and the fruit fly clock is particularly sensitive to blue light, with cryptochrome (CRY) as its primary photoreceptor (Emery et al., 2000). Therefore, we expected that transitioning from white/dark to blue/dark lighting patterns would have an acute wake-promoting effect during exposure. Our results support this hypothesis, as blue light disrupts sleep and has an acute arousal effect in wild-type flies of both sexes (Figures 2–4). This acute arousal could act as an adaptive response, signaling the animal to initiate locomotion and avoid the high-frequency irradiation. This wavelength-specific behavioral plasticity aligns with findings from Lazopulo et al. (2019), who demonstrated that flies have a daytime-specific aversion to blue light. Exposure to blue light generally has negative effects on the health and lifespan of flies, including neurodegeneration, likely through oxidative stress (Arthaut et al., 2017; Hall et al., 2018; Krittika and Yadav, 2022; Nash et al., 2019). Thus, adaptive behavioral responses to avoid the adverse effects of short-wavelength light could provide a fitness advantage.

Daytime behavioral aversion to blue light in flies is mediated by Rhodopsin 7 (Rh7) and CRY, the two internal blue-light photoreceptors in the *Drosophila* brain—both of which can entrain the circadian clock and are mediate some behavioral responses to UV light, another form of short-wavelength light (Baik et al., 2019). Blue light-induced CRY activation in the l-LNvs, master clock cells in the fly brain, stimulates release of the wake-promoting neuropeptide pigment-dispersing factor (PDF), which is essential for light-based arousal (Parisky et al., 2008; Shang et al., 2008). CRY and Rh7 are attractive molecules as mediators for the sleep response to blue light. However, blue-light input pathways in the compound eye also exist, as Rh4, Rh5, and Rh1 also respond to blue wavelengths (Sharkey et al., 2020). Future studies should aim to determine which specific photoreceptors are involved in blue light’s regulation of sleep.

The acute arousing effect of blue light we observed differs from findings by Krittika and Yadav (2022), who noted that flies exposed long-term to a 12:12 BD cycle slept more during the day than those in a traditional WD condition. Our 12:12 BD condition led to acute reductions in sleep (relative to baseline) among CS flies. These conflicting results may stem from distinctions in experimental design—our study used flies as their own controls by assessing baseline sleep in WD before switching to BD (“within-subjects design”), as opposed to comparing between two sets of test groups. On a related note, Krittika and Yadav (2022) experimental design included a more chronic BD photoperiod (10 d) that began immediately post-eclosion, and data from all 10 d were averaged together, so the elevated daytime sleep they observed in BD could have been a progressive compensatory response against chronic light-induced cellular stress, as sleep has an intimate relationship with the restoration of oxidative stress (Haynes et al., 2024; Hill et al., 2018; Vaccaro et al., 2020). Unlike prior studies (Krittika and Yadav, 2022; Shen et al., 2021), we did not note any differences in mortality across light-color experiments, with most rates < 10%. This lack of effect may stem from our shorter experimental timeline of only 6 d of light-color treatment, which was much shorter than the exposure period in the other studies mentioned.

In addition to a daytime sleep reduction during the exposure, we also hypothesized that blue light would impact nighttime sleep (after “lights off”). Our results also support this hypothesis, as we noted that 12 h of blue light profoundly reduces nighttime sleep on the second and third days of exposure in CS flies (Figure 2). Interestingly, this decrease in sleep was only observed with 12 h of treatment, as neither 3-h morning nor evening exposures was sufficient to elicit the same effect (Figures 3, 4). Surprisingly, evening exposure to blue light increased nighttime sleep in CS flies (Figure 4), suggesting that a 3-h evening exposure to blue light is insufficient to model the sleep effects of VDU exposure on humans, and instead, 12 h of blue light is needed to elicit robust nighttime sleep reductions in wild-type flies.

### 4.2 Absent blue light effects in *white* mutants provide mechanistic insights

We have consistently found that *w*^1118^ flies show no daytime sleep inhibition from blue light (Figures 2–4). Our *w*^1118^ flies possess the *w*^1118^ allele, a null mutation in *white*, the first gene identified in *Drosophila* (Morgan, 1910). *White* encodes an ATP binding cassette (ABC) transporter that dimerizes with either Scarlet or Brown to traffic metabolic precursors throughout the animal, with highest expression in the compound eye (Ferreiro et al., 2017). The White-Scarlet dimer transports tryptophan to the eye and through the brain, while White-Brown carries guanine (Ferreiro et al., 2017). In the compound eye, guanine and tryptophan serve as precursors to drosopterin and ommochrome—the two pigments which give wild-type flies their brick-red appearance (Tomlinson, 2012). The primary function of these screening pigments is to improve visual acuity by maintaining photons within each ommatidium, so they do not activate photoreceptors in other nearby cells (Tomlinson, 2012). They may also protect photoreceptors from damage associated with excessive light exposure, as *white* mutants exhibit signs of retinal degeneration at just 5 days old (Ferreiro et al., 2017). White-transported guanine and tryptophan are essential precursors in the synthesis of biogenic amines, such as serotonin, dopamine, octopamine, and histamine; consequently, *white* mutants exhibit especially low levels of histamine in the brain (Borycz et al., 2008). Intriguingly, histamine is a primary neurotransmitter used by the *Drosophila* visual system, which could underlie *w*^1118^ flies’ lack of an acute reaction to blue light. In other words, *w*^1118^ flies may sense the blue light in the compound eye but then be unable to transmit this information to the central brain due to histamine deficiency. Ultimately, the lack of a daytime sleep-inhibiting effect of blue light in *w*^1118^ flies suggests that the compound eye and downstream pathways may play a role in mediating the sleep effects of blue light. This would fit with a recent report demonstrating that neither just the compound eye nor CRY is sufficient for blue light-induced l-LNv activity (Au et al., 2023). Both pathways (and Rh7) are also important for acute arousal from sleep with a blue light pulse, with an especially outsized role for Rh7 at higher light intensities (Au et al., 2023). However, it has also been shown that other neurotransmitter imbalances caused by mutations to the *white* gene, can be behaviorally relevant (Alekseyenko and Kravitz, 2014; Kain et al., 2012; Sitaraman et al., 2008). Thus, those changes could affect sleep independently of effects on function of the compound eyes.

As with daytime effects of blue light, *w*^1118^ flies did not exhibit the same nighttime sleep reduction that was observed in CS flies (Figure 2), possibly implicating the compound eye in this nighttime sleep suppression as well. As the sleep loss in CS flies was spread across the night (Figures 2B, C), it appeared that it was not a defect in nighttime sleep onset, but rather in sleep maintenance. Interestingly, visual experience regulates sleep need in flies, as complex visual stimuli increase nighttime sleep consolidation (Kirszenblat et al., 2019), but any role for light color in regulating sleep need has yet to be elucidated. Nighttime sleep maintenance in *Drosophila* is regulated through a myriad of signals, including NPF release and GABAergic inhibition of the PDFergic l-LNvs (Gmeiner et al., 2013; He et al., 2013). l-LNvs are activated by CRY phototransduction but also by compound eye inputs (Au et al., 2023; Schlichting et al., 2016), which we expect to be involved in this given absent effect in *w*^1118^. Like with daytime sleep, the lack of effect in *w*^1118^ flies could result from disrupted phototransduction due to the absence of screening pigment or neurotransmitter monoamines downstream from photoreception.

To validate the compound eye’s role in mediating these blue light’s effects on both daytime and nighttime sleep, future studies could examine how a 12-h blue light exposure influences sleep in *glass* or *norpA* mutants. *Glass* encodes a transcription factor critical for cell-fate decisions underlying photoreceptor identity in the developing retina and HB eyelet, rendering *glass* mutants completely reliant on internal sensors like CRY to entrain to environmental light (Bernardo-Garcia et al., 2016; Helfrich-Forster et al., 2001). On the other hand, *norpA* encodes a phospholipase-Cβ involved in rhodopsin signal transduction in compound eye photoreceptors, leading *norpA* mutants to also have dysfunctional vision and altered photoentrainment (Bloomquist et al., 1988; Breda et al., 2020).

### 4.3 Red light-induced time of day-specific sleep reductions

**FIGURE 11:**
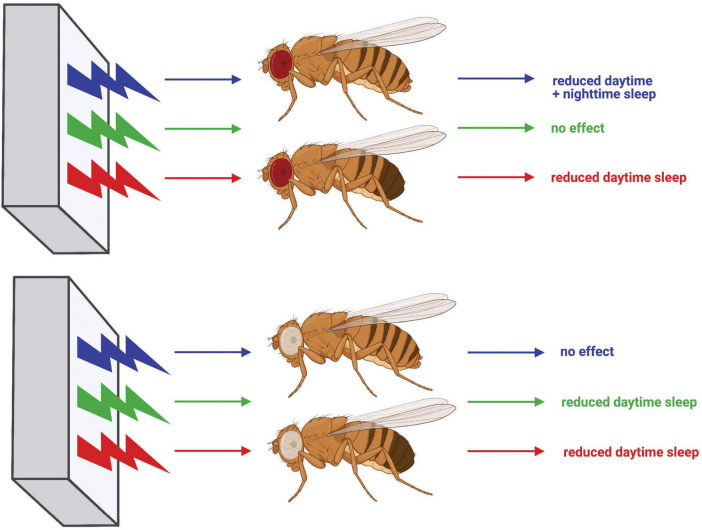
Diagram summarizing the effects of light color on sleep in CS and *w*^1118^ flies. Effects of exposure to blue, green, and red light on red-eyed CS flies are shown at the top, whereas effects on white-eyed *w*^1118^ flies are shown at the bottom. Schematic generated using BioRender.com.

Perhaps an even stronger effect than what was observed with blue light was the daytime sleep suppression induced by red light (Figures 8–11). With 12 h of red light, we observed a consistent daytime sleep reduction not only across sexes, but also in both CS and *w*^1118^ flies (Figure 8). Moreover, CS and *w*^1118^ flies returned to WD sleep levels during the recovery period, ruling out the possibility that sleep changes earlier in the experiment were merely developmental (Figures 8, 11). Lower daytime sleep in the presence of red light aligns with findings from Krittika and Yadav (2022), but our effects reached a statistically significant level. Along with reduced evening (ZT9–12) sleep, an overall phase advance in sleep patterns was noted due to both 12 h and evening red-light exposures (Figures 8, 10). Despite this phase advance, CS flies did maintain rhythmicity, corroborating prior evidence that wild-type *Drosophila* can entrain to red light (Hanai et al., 2008; Zordan et al., 2001). Ethologically, the evening wake-promoting effect of red light may represent a behavioral adaptation of *Drosophila* to natural daily changes in sunlight’s spectral composition. While present across the whole day, long wavelengths like red light are especially enriched at sunset (Hut et al., 2000; Moon et al., 2016). Thus, the crepuscular *Drosophila* may have evolved to coordinate its evening activity with red-enriched light at sunset, such that greater ambient red-light composition acts as a time-of-day cue to the animal to awaken from its midday rest and pursue food, mates, etc.

We also noted that the evening 3-h exposure to red light potently inhibited sleep during the exposure in both CS and *w*^1118^ flies (Figure 10). The arousal response to a low-intensity pulse of red light has been shown to be dependent on the compound eye (Au et al., 2023). In our study, a similar acute sleep suppression was noted in *w*^1118^ flies with an evening exposure to green light (Figure 7). Indeed, *w*^1118^ males also showed reduced daytime sleep in the presence of 12 h of green light, with peak sleep loss occurring in the evening (Figure 5). Taken together, these findings suggest that the same downstream sleep effects elicited by evening exposure to red light in CS flies may be elicited by green light in *w*^1118^. Sharkey et al. (2020) demonstrated that the presence of screening pigment shifts the spectral sensitivity of particular rhodopsins in the compound eye, which may account for the similar effects of green light on *w*^1118^ and red light on CS. Specifically, Rh6 shows peak sensitivity to 600-nm (red) light in red-eyed flies but to 510 nm (green) in white-eyed flies. Importantly, Rh6 along with Rh1 mediate circadian entrainment to red light (Hanai et al., 2008). Upcoming studies will directly test our hypothesis that *Rh6* is essential for the acute sleep effects of evening red or green light in CS and *w*^1118^ animals, respectively. The evening-specific nature of this effect could imply the existence of a sleep-regulating pathway in which Rh6-dependent phototransduction (via either the compound eye or the Rh6+ HB eyelet) sits upstream of E cells, the clock neuron subtype in the fly brain responsible for controlling the circadian activity rhythms during the evening (Brown et al., 2024).

### 4.4 Limitations

It is worth noting that experiments presented in Figures 2–10 represent one replicate of each experiment; however, our main results closely resemble several additional renditions of the same experiments from our lab, using either the same light delivery system or completely distinct lighting units—underscoring the reliability and reproducibility of our light color-specific findings. For example, nighttime sleep reductions after 12 h blue light reliably occur on days 2–3 of exposure regardless of light delivery system. Moreover, slight differences in light intensity between the white light and light color conditions could account for some effects, but unpublished results from our lab show that altering light intensity alone has negligible impact on sleep. Another potential limitation to our study design is the difficulty to ascertain if effects across days are due to the light color exposure or simply aging-associated changes. Considering that sleep behavior largely stabilizes at 3 d post-eclosion (Shaw et al., 2000), and all flies tested were ≥ 3 d old, the latter appears unlikely. Further, the transience and specificity of our sleep effects also disputes this interpretation. For example, if blue light-associated nighttime sleep reductions observed in Figure 2 were strictly due to age, one would expect the effect to persist and progress as the experiment continued. Rather, this effect largely disappears after ∼2 d. Lastly, the nature of our experimental design makes it difficult to assess if light color-specific sleep phenomena are directly caused by the light color being emitted or rather by the absence of others. For example, are daytime sleep reductions in RD conditions due to the red light itself or the absence of blue-green input? Studies that just remove one of these light color inputs (e.g., only green-red input without blue) could help answer this question. Nevertheless, our results unequivocally indicate that light color is an important environmental modulator of fruit fly sleep behavior.

## 5 Conclusion

Taken together, our results illustrate that fruit fly sleep, much like human sleep, is subject to light color-specific regulation (Figure 11), and demonstrate time-of-day dependency for the effects of specific light colors on sleep. This work further establishes *D. melanogaster* as an effective model to study how environmental cues are received by the brain and integrated to facilitate complex behavior. Moreover, our within-subject experimental design provides the first direct evidence of light color-dependent sleep/wake behavioral plasticity in flies. Researchers commonly use red or blue light to optogenetically activate specific neuronal populations in *Drosophila*. However, our findings underscore that experimentation combining optogenetics with behavioral analysis must be interpreted knowing that the optic stimulus may elicit behavioral changes, independent of the transgenic light-gated ion channel. Additionally, white-eyed *w*^1118^ flies are often employed as a “wild-type” strain in sleep studies, but our results indicate a substantially different behavioral response to blue light as compared with red-eyed CS flies. This finding should caution the use of *white* mutants as wild-type animals when investigating the interplay between environmental cues and sleep behavior. These findings in *w*^1118^ flies additionally provide mechanistic insight into how light color affects sleep, pointing to transmission of light color information from the compound eye to central brain sleep circuits. Future experiments should seek to elucidate the logic behind how the fly brain encodes light color information in these sleep circuits and how that code is translated into behavior. Ultimately, further work employing *Drosophila* to study mechanisms surrounding light color’s sleep effects will undoubtedly provide novel insights into how the environment modulates neural circuits to effect behavioral change and may lead to improved technologies to combat the negative impacts of blue light-enriched screen use.

## Data Availability

The raw data supporting the conclusions of this article will be made available by the authors, without undue reservation.
